# The effects of illness perceptions, self‐efficacy and mental wellbeing on uptake and completion of a diabetes prevention programme in England

**DOI:** 10.1111/bjhp.70086

**Published:** 2026-07-13

**Authors:** Sonia Begum, Rachel Povey, Paul Chadwick, Naomi Ellis, Christopher Gidlow

**Affiliations:** ^1^ School of Psychology and Counselling The Open University Milton Keynes UK; ^2^ Arden University Birmingham UK; ^3^ School of Health, Education, Policing and Sciences University of Staffordshire Stoke‐on‐Trent UK; ^4^ Centre for Behaviour Change University College London London UK; ^5^ Centre for Health and Development University of Staffordshire Stoke‐on‐Trent UK; ^6^ School of Medicine Keele University University Road Staffordshire UK; ^7^ Midlands Partnership University NHS Foundation Trust, Research and Innovation Department St Georges Hospital Stafford UK

**Keywords:** completion, diabetes prevention, illness perceptions, mental wellbeing, self‐efficacy, uptake

## Abstract

**Introduction:**

Psychological factors, such as illness perceptions, self‐efficacy and mental wellbeing, can influence uptake and completion of health programmes. Whether these psychological factors predict uptake and completion of diabetes prevention programmes is not currently known. This study explored whether these modifiable psychological factors predict uptake and/or completion of the Healthier You: National Health Service Diabetes Prevention Programme in England, independent of non‐modifiable factors.

**Methods:**

Data were collected by a local provider as part of their service evaluation, using convenience sampling whereby those who attended an initial assessment were asked to complete psychological measures. Binomial logistic regression analyses were conducted to identify whether illness perceptions, self‐efficacy and mental wellbeing predicted uptake and/or completion, independent of region, age, gender, ethnicity and deprivation.

**Results:**

Three thousand seven hundred and fifty‐six participants with prediabetes from England were included in the analyses. Illness perceptions (treatment control‐odds ratio (OR) = 1.03; confidence interval (CI) = 1.01–1.06; *p* = .006 and illness concern‐ OR = 1.06; CI = 1.04–1.09; *p* = <.001), mental wellbeing (OR = 1.18; CI = 1.01–1.39; *p* = .042), age and deprivation were significant predictors of uptake. Self‐efficacy (OR = .97; CI = .96–.99; *p* = <.001), age and region were significant predictors of completion.

**Conclusions:**

This study demonstrates the importance of modifiable psychological factors, particularly illness perceptions and mental wellbeing, in predicting uptake and self‐efficacy predicting completion of a diabetes prevention programme. Early assessment of these psychological factors should be undertaken to identify individuals at risk of not starting or not completing diabetes prevention programmes so uptake and retention can be optimized.

**Statement of Contribution**

**What is already known on this subject?**

- Although diabetes prevention programmes have been found to be effective, there is limited uptake of these programmes and even lower completion rates.
- Psychological factors, such as illness perceptions, self‐efficacy and mental wellbeing, can influence uptake and completion of health programmes but there is lack of evidence as to whether these factors can predict uptake and completion of diabetes prevention programmes.

**What does this study add?**

- Illness perceptions related to treatment control and illness concern were significant predictors of uptake.
- Mental wellbeing was a significant predictor of uptake.
- Self‐efficacy was a significant predictor of completion.

## INTRODUCTION

Type 2 diabetes prevention is a global health care priority (International Diabetes Federation, 2021; Saeedi et al., 2019). Levels of prediabetes (i.e., those at high risk of type 2 diabetes) are predicted to rise from 374 million (in 2019) to 548 million by 2045 (Saeedi et al., 2019). In England, it is forecasted that 1 in 10 people will have type 2 diabetes by 2034, highlighting the need for more preventative measures (Public Health England, 2014). The successful implementation of diabetes prevention programmes in countries like Finland (Lindstrom et al., 2006), has led England to develop a lifestyle behaviour change programme, the Healthier You: National Health Service Diabetes Prevention Programme (NHSDPP) (Valabhji et al., 2020).

It is important that diabetes prevention programmes are clinically effective and financially viable when delivered at scale. Programme viability is dependent on reaching and retaining adequate numbers of the target population, that is, uptake and retention (Alva, 2019). Uptake is defined as participants attending the first or at least one programme session, and retention refers to sustained participant involvement, ideally until programme completion (NHS England, 2016; Valabhji et al., 2020). The NHSDPP (2016–2018) found 63%–69% of individuals who attended the initial assessment, a short appointment they are required to attend before programme commencement, attended at least one session (i.e., ‘uptake’) (Howarth et al., 2020; Valabhji et al., 2020). Between 2016 and 2018, completion rates for the NHSDPP have been reported as 19–22% (Howarth et al., 2020; Valabhji et al., 2020). By understanding the factors involved that affect participants' decisions as to whether to start or complete a programme, uptake and retention can be further improved.

It is important for diabetes prevention programmes to identify both non‐modifiable and modifiable risk factors like psychological factors (e.g., the way people think about their condition and their ability to change), that may affect uptake and/or completion (Aujla et al., 2019; Valabhji et al., 2020). Illness perceptions, self‐efficacy and mental wellbeing are important factors for diabetes prevention programmes to consider as they have been identified as influencing uptake and/or completion for other health programmes, and can be targeted to improve uptake and retention rates (Broadbent et al., 2006; Cassidy et al., 2014; Deskur‐Śmielecka et al., 2009; French et al., 2006; Jancey et al., 2007; Kampshoff et al., 2016; Khalil et al., 2012; Marmarà et al., 2017; Murray et al., 2012; Selzler et al., 2019; Whitmarsh et al., 2003; Yohannes et al., 2007).

Illness perceptions have been derived from the Common‐Sense Model of Self‐Regulation (CSM) (Leventhal, 1970; Leventhal et al., 1980, 1992). This is a health‐specific model which provides a theoretical basis to allow individuals' emotional and cognitive responses to a health threat to be explored (Leventhal et al., 1980). Illness perceptions are an important component of this model and are beliefs or cognitive perceptions held by individuals regarding their illness (Petrie et al., 2007). These illness perceptions include illness *consequences* (perceptions held on the expected effects of an illness), *timeline* (perceptions held on the duration of an illness), *personal control* (how much an individual feels they have control over their illness), *treatment control* (perceptions held on the extent to which treatment can help to control an illness), *identity* (perceptions held on the label and symptoms of an illness), *coherence* (perceptions held on the understanding of an illness), *concern* (the extent to which how worried an individual feels about their illness), *emotional response* (perceptions held on the emotional effects of an illness) and *causes* (perceptions held on the cause of an illness) (Broadbent et al., 2006; Moss‐Morris et al., 2002; Petrie & Weinman, 2006). These illness perceptions are vital in influencing coping strategies and specific behaviours related to the illness such as diabetes treatment adherence (Petrie & Weinman, 2006). The CSM can be used to understand treatment engagement. Individuals generate cognitive representations and emotional responses when they are faced with a health threat or illness and are trying to make sense of these threats or illnesses (Leventhal et al., 1992). To self‐regulate, individuals will make efforts to find ways to manage these cognitions and emotions (Leventhal et al., 1992). These efforts will result in individuals participating in ‘common‐sense’ health behaviours such as seeing a doctor, taking medication or attending a diabetes prevention programme that is offered to them after diagnosis (Leventhal et al., 1992).

Research exploring predictors of uptake in other health preventative and lifestyle behaviour change programmes has demonstrated that certain illness perceptions significantly predict higher uptake (Marmarà et al., 2017, 2019; Murray et al., 2012). Studies have shown that attenders (i.e., those who started a programme) had higher illness identity or controllability scores than non‐attenders (Broadbent et al., 2006; Cooper et al., 1999; French et al., 2006; Petrie et al., 1996; Whitmarsh et al., 2003). Also, participants with more positive identity, more control, who believed there were worse consequences, and had coherent beliefs were more likely to attend, with controllability being the strongest predictor of attendance than the other illness perception components (French et al., 2006). Non‐completion has been associated with a lower number of illness consequences and lower perceptions of controllability or treatment control (Whitmarsh et al., 2003; Yohannes et al., 2007), as well as higher levels of personal control (Yohannes et al., 2007) in two studies of cardiac rehabilitation programmes. However, whether illness perceptions influence uptake and/or completion for diabetes prevention programmes remains unknown.

Self‐efficacy is another important factor for diabetes prevention programmes to consider. General self‐efficacy relates to beliefs in one's overall capacity to perform tasks (Smith et al., 2010), and an efficacy expectation has been defined as ‘the conviction that one can successfully execute the behavior required to produce [target] outcomes’ (Bandura, 1977, p. 193). Self‐efficacy is an important link between knowledge application and actual behaviour change, and it is one of the key, effective predictors of health behaviour (Bandura, 1982; Chen & Lin, 2010). Although many diabetes prevention programmes have demonstrated that increased self‐efficacy (as a result of the programme) has led to improvements in physical activity (Block et al., 2016; Cioffi et al., 2018; Leung et al., 2019), healthy eating (Block et al., 2016; Leung et al., 2019), weight loss (Delahanty et al., 2013; Hays et al., 2014) and reduction in the risk of developing type 2 diabetes by promoting healthier lifestyles (Chen & Lin, 2010), it is not clear whether self‐efficacy predicts the uptake and/or completion of diabetes prevention programmes. Research on other behaviour change programmes, such as cardiac rehabilitation and lifestyle behaviour change programmes, has shown that self‐efficacy was related to attendance (Murray et al., 2012; Selzler et al., 2019), and those with lower levels of self‐efficacy were more likely to become non‐completers (Jancey et al., 2007; Kampshoff et al., 2016). However, whether self‐efficacy predicts uptake and/or completion of diabetes prevention programmes like the NHSDPP is unknown.

Mental wellbeing refers to positive mental health, which includes the subjective experience of happiness and life satisfaction, positive psychological functioning and developing and sustaining good personal and social relationships (Stewart‐Brown & Janmohamed, 2008). Studies have shown that mental wellbeing can affect the uptake and attendance of lifestyle behaviour change (Khalil et al., 2012; Murray et al., 2012) and cardiac rehabilitation programmes (Deskur‐Śmielecka et al., 2009). Some have also indicated that mental wellbeing can positively affect completion or encourage continued attendance (Cassidy et al., 2014; Khalil et al., 2012), whereas others have found that those with post‐traumatic stress disorder and/or depression were more likely to participate in cardiac rehabilitation compared to those who did not have these conditions (Krishnamurthi et al., 2019). Although some diabetes prevention programmes have found that as a result of programme participation, mental wellbeing improved or self‐esteem increased (Castro Sweet et al., 2018; Kulzer et al., 2009; Quiñones et al., 2018), and others have demonstrated that completers had better mental wellbeing (lower scores on measures of psychological distress, anxiety and depression) when compared to non‐completers (Laatikainen et al., 2007; Teuschl et al., 2012), it remains unclear as to whether mental wellbeing predicts uptake and/or completion of a diabetes prevention programme like the NHSDPP. Overall, the literature suggests people with poor mental wellbeing may be less likely to attend due to the characteristics of low mood and higher levels of anxiety, but further exploration of this is needed. It would be useful to know whether mental wellbeing affects programme attendance, in order to target the interventions appropriately.

To date, there is a lack of literature on the effects of psychological factors on the uptake and/or completion of diabetes prevention programmes. This information can help diabetes prevention programmes improve recruitment and retention by adapting recruitment methods and tailoring intervention components to meet patient needs. This study aimed to explore whether illness perceptions, self‐efficacy and mental wellbeing predict uptake and/or completion of the NHSDPP in England, independent of other non‐modifiable confounders (region, age, gender, ethnicity and deprivation).

The research questions were:

- Do certain pre‐programme illness perceptions predict uptake and/or completion of diabetes prevention programmes?
- Do pre‐programme self‐efficacy scores predict uptake and/or completion of diabetes prevention programmes?
- Do pre‐programme levels of mental wellbeing significantly predict uptake and/or completion of diabetes prevention programmes?

In line with the current literature, specific hypotheses were as follows:

1. Certain pre‐programme illness perceptions will predict uptake and completion, with illness treatment control being the most salient. Participants with higher treatment controllability scores will be more likely to attend and complete the programme.
2. Participants with lower pre‐programme self‐efficacy scores will be less likely to attend or complete the programme.
3. Participants with higher (i.e., better) pre‐programme mental wellbeing scores will be more likely to complete the programme.

## MATERIALS AND METHODS

### The local provider diabetes prevention Programme

At the time of the study, the NHSDPP was delivered by four providers under a framework agreement with NHS England (Valabhji et al., 2020). This study used service evaluation data from one of these providers. The programme consisted of 18 sessions delivered over 9 months and covered a variety of different topics on nutrition, physical activity and behaviour change (Macmillan, 2016). Potential participants were referred to the programme from primary care and invited to an initial assessment. Those referred were identified as having prediabetes after undergoing an NHS Health Check, through routine clinical practice or by obtaining qualifying blood test results via GP records (Barron et al., 2018). The initial assessment aimed to establish eligibility, allow participants to learn more about the intervention and take baseline measurements (Macmillan, 2016).

### Design and participant selection

Data were collected by the local provider as part of their service evaluation, and ethical approval was then obtained from the University of Staffordshire. Data were collected routinely by the local provider from 10,739 participants attending initial assessments in various areas of England (April 2016 to January 2018), with informed consent obtained. Convenience sampling was used, whereby those who attended the initial assessment were asked to complete measures of illness perceptions, self‐efficacy and mental wellbeing. All participants who were eligible to take part in the study were registered with the local provider, had attended the initial assessment and were adults identified as having non‐diabetic hyperglycaemia (also known as prediabetes).

### Data collection and variables

At the initial assessment, a range of data were collected for each participant including:

- *Demographic*: Age, gender, ethnicity, region and postcode were recorded. Deprivation was calculated using the index of multiple deprivation, derived from participant postcode.
- *Illness perceptions*: The nine‐item Brief Illness Perception Questionnaire (Brief IPQ) was used to measure IPs, with the term ‘illness’ replaced by ‘prediabetes’ (i.e., the illness of interest) as recommended (Broadbent et al., 2006). Illness perception scores were taken for the following items of the Brief‐IPQ: consequences, timeline, personal control, treatment control, identity, coherence, concern and emotional response (Broadbent et al., 2006). For each item, participants were asked to rate how they felt about a certain question using a Likert scale and selected a score from 0 to 10.
- *Self‐efficacy*: The eight‐item New General Self‐Efficacy Scale (NGS‐ES) (Chen et al., 2001) was used to measure general self‐efficacy, calculated as a summary score (Smith et al., 2010). For each item, participants were asked to rate how they felt about a certain statement using a 5‐point Likert rating scale (1 = strongly disagree; 3 = neither agree nor disagree; 5 = strongly agree).
- *Mental wellbeing*: The 14‐item Warwick‐Edinburgh Mental Wellbeing Scale (WEMWBS) (Stewart‐Brown & Janmohamed, 2008) was used to measure mental wellbeing. The total score was calculated and categorized as low (14–42), medium (43–59) or high (60–70) in accordance with guidelines (University of Warwick, 2020). This avoided violating assumptions of linearity of the logit in logistic regression. For each item, participants were asked to respond to a certain statement using a 5‐point Likert rating scale (1 = none of the time; 3 = some of the time; 5 = all of the time).
- *Uptake and completion*: Those who attended the first session (or at least one session) of the NHSDPP following their initial assessment were classified as attenders (i.e., successful uptake). Those who attended at least 75% of sessions (14/18) were classified as completers, in line with the criterion definition used by NHS England for payment by completion (NHS England, 2016). This led to dichotomous outcome variables for uptake (yes/no) and completion (yes/no).

### Statistical analysis

Data were checked for normal distribution, errors and extreme values. Binomial logistic regression models were used to explore predictors of uptake and completion, entering variables sequentially. Demographic variables (age, gender, ethnicity, region and deprivation) were included in step one, illness perception items in step two, general self‐efficacy total scores in step three and mental wellbeing scores in step four. The demographic variables were entered first to see if they predicted uptake and/or completion irrespective of the psychological variables. The psychological variables were entered thereafter to see whether they predicted uptake and/or completion independent of the demographic variables. Our ultimate interest was whether or not the illness perception items, general self‐efficacy and mental wellbeing were associated with uptake or completion, independent of participant socio‐demographics, which were entered in step 1.

Models were tested for assumptions of linearity, independence of errors and multicollinearity. Assumptions of linearity were tested for all three questionnaires (i.e., using the scores of the illness perception items, general self‐efficacy and mental wellbeing). Following exploration of data transformations, variables were categorized as detailed in the data collection and variables section. The significance level used was .05. To determine which variables to include in logistic regression, Mann–Whitney *U* Tests were conducted to explore differences between illness perception item scores for participants who took up the programme/completed the programme and those who did not (Appendix S1). As a result of these tests, only those illness perception items that were found to show significant differences for uptake (consequences, timeline, treatment control and illness concern) and for completion (consequences and personal control) were included in the logistic regression models respectively. Participants who had complete outcome data were included in the regression, and those who did not were excluded. Mann–Whitney *U* and Multi‐Dimensional Chi‐Square Tests were conducted to determine whether participants included in the regression versus those excluded from the regression for uptake differed in terms of key characteristics (Appendix S2). The Omnibus Tests of Model Coefficients were used to decide the model fit of the logistical regression models.

Multiple imputation was conducted to investigate the effect of missing data. Binomial logistical regression analyses were repeated using imputed data sets and these results were compared to the original analyses. SPSS version 27 was used for data analyses.

## RESULTS

### Demographic and clinical characteristics

From the available data (before analyses), 5387 individuals started the NHSDPP (56.9% uptake, excluding missing data), and 791 participants completed the programme (14.7% completion). Out of the 10,739 individuals referred to the NHSDPP who attended the initial assessment across six areas, outcome data were available for 3756 participants, which were used in the logistic regression, the data for whom are summarized in Table 1.

**TABLE 1:** Sample characteristics.

	Included in LR for uptake (*n* = 3756)	Included in LR for completion (*n* = 2344)
	Frequency (%)	Frequency (%)
Participant characteristics		
Gender		
Women	2047 (54.5)	1318 (56.2)
Men	1709 (45.5)	1026 (43.8)
Age		
Mean (SD)	62.5 (12.5)	64.1 (11.7)
<40	166 (4.4)	65 (2.8)
40–44	166 (4.4)	74 (3.2)
45–49	245 (6.5)	126 (5.4)
50–54	405 (10.8)	236 (10.1)
55–59	480 (12.8)	292 (12.5)
60–64	473 (12.6)	289 (12.3)
65–69	609 (16.2)	416 (17.7)
70–74	583 (15.5)	419 (17.9)
≥75	629 (16.7)	427 (18.2)
Ethnicity		
White British/White	2378 (63.3)	1571 (67.0)
Black	701 (18.7)	414 (17.7)
Asian	505 (13.4)	260 (11.1)
Mixed	96 (2.6)	66 (2.8)
Other	76 (2.0)	33 (1.4)
Region		
South London	1627 (43.3)	992 (42.3)
North East London	354 (9.4)	174 (7.4)
Berkshire	569 (15.1)	331 (14.1)
Cumbria	548 (14.6)	398 (17.0)
Herefordshire	451 (12.0)	297 (12.7)
West Yorkshire	207 (5.5)	152 (6.5)
Deprivation quintile		
1 (most deprived)	865 (23.0)	492 (21.0)
2	899 (23.9)	509 (21.7)
3	801 (21.3)	499 (21.3)
4	579 (15.4)	397 (16.9)
5 (least deprived)	612 (16.3)	447 (19.1)
Uptake		
Yes	2263 (60.3)	‐
No	1493 (39.7)	‐
Number of completers		
Yes	‐	515 (22.0)
No	‐	1829 (78.0)

Abbreviation: LR, binomial logistic regression.

The only statistically significant differences between the 3756 participants included in the logistic regression for uptake and the 6983 excluded due to missing data were for region, mental wellbeing and the illness perception item timeline (Appendix S2). There was a significant but weak association between region and those excluded and included in the logistic regression (*Χ* ^2^ (5, *N* = 10,739) = 643.178, *p* = <.001) (Appendix S2).

There was a significant, but weak association between mental wellbeing and those excluded and included in the logistic regression (*Χ* ^2^ (2, *N* = 10,679) = 11.601, *p* = .003; *ϕ* = .033) (Appendix S2). Those participants who were included in the logistic regression had slightly higher proportions of medium mental wellbeing and slightly lower proportions of high or low wellbeing compared with those excluded; however, the differences were minimal. The illness perception scores for timeline were not significantly different in those included and excluded in the logistic regression for uptake (Median = 3.00) (Appendix S2).

Overall, despite statistically significant associations between region/mental wellbeing and those excluded and included in the logistic regression, the associations were weak and the differences minimal. Therefore, included and excluded participants were considered sufficiently similar for results to be representative of the full sample.

A total of 2344 participants were included in the logistic regression analysis for completion out of a total of 3756 (Table 1). For both uptake and completion, large sample sizes were included in the logistic regression models (*n* = 3756 for uptake, *n* = 2344 for completion). By following published guidelines for determining the minimum sample size needed for logistic regression analyses (Bujang et al., 2018), this study had sufficient data to produce reliable estimates/results. Based on the recommended calculation of *n* = 100 + (50 x number of IVs), the minimum sample required was 650 (for uptake) and 550 (for completion).

### Predictors for uptake

Data were checked for independence from errors. For uptake, the chi‐squared goodness‐of‐fit statistic showed there was no overdispersion (*χ* ^2^ (8): 4.844, *p* = .774). There was no evidence of collinearity between the predictor variables. Assumptions of linearity between the continuous predictors (illness perceptions, general self‐efficacy) and the logit of the outcome variable were met. The region with the lowest uptake was used as the reference category (North East London: 51.2%, *n* = 608). Overall, the omnibus tests of model coefficients showed the model had a good fit against the null model (*χ* ^2^ (29): 223.931, *p* = <.001) but explained just 8% of variance (Nagelkerke *R* ^2^ = .078).

Higher scores on the illness perception item relating to treatment control (view treatment as being effective in controlling their prediabetes) and illness concern (more concerned about their prediabetes) were associated with significantly increased odds of starting the NHSDPP (treatment control OR = 1.03; CI = 1.01–1.06; *p* = .006; illness concern OR = 1.06; CI = 1.04–1.09; *p* = <.001) (Table 2). Compared with individuals with high mental wellbeing scores, those with medium mental wellbeing scores (moderate mental wellbeing) had an 18% higher odds of starting (OR = 1.18; CI = 1.01–1.39; *p* = .042). There were no associations between uptake and low mental wellbeing or general self‐efficacy.

**TABLE 2:** Results of the binomial logistic regression model exploring predictors of uptake of the NHSDPP.

	Step 1		Step 2		Step 3		Step 4	
	OR (95% CI)	*p*	OR (95% CI)	*p*	OR (95% CI)	*p*	OR (95% CI)	*p*
Region [North East London]		.001		<.001		<.001		<.001
Cumbria	1.56 (1.13–2.15)	.007	1.68 (1.21–2.33)	.002	1.67 (1.21–2.32)	.002	1.69 (1.22–2.34)	.002
Herefordshire	1.18 (0.84–1.65)	.334	1.23 (0.88–1.72)	.237	1.21 (0.86–1.70)	.269	1.21 (0.86–1.71)	.265
Berkshire	0.92 (0.68–1.26)	.611	0.95 (0.70–1.30)	.766	0.95 (0.69–1.29)	.724	0.96 (0.70–1.31)	.790
South London	1.15 (0.89–1.48)	.284	1.15 (0.89–1.49)	.281	1.14 (0.88–1.48)	.319	1.15 (0.89–1.49)	.299
West Yorkshire	1.61 (1.08–2.40)	.018	1.80 (1.20–2.69)	.004	1.80 (1.20–2.69)	.004	1.80 (1.20–2.69)	.004
Gender [Male]: Female	1.14 (1.00–1.31)	.055	1.09 (0.95–1.25)	.204	1.09 (0.95–1.25)	.204	1.08 (0.94–1.24)	.259
Age [<40]		<.001		<.001		<.001		<.001
40–44	1.19 (0.77–1.86)	.435	1.23 (0.79–1.91)	.372	1.23 (0.79–1.92)	.368	1.22 (0.78–1.91)	.380
45–49	1.55 (1.03–2.33)	.034	1.59 (1.05–2.39)	.027	1.59 (1.05–2.39)	.028	1.59 (1.05–2.39)	.028
50–54	2.03 (1.39–2.96)	<.001	2.05 (1.40–3.01)	<.001	2.05 (1.40–3.01)	<.001	2.05 (1.40–3.00)	<.001
55–59	2.09 (1.44–3.04)	<.001	2.11 (1.45–3.07)	<.001	2.11 (1.45–3.07)	<.001	2.10 (1.45–3.06)	<.001
60–64	2.00 (1.38–2.92)	<.001	2.09 (1.43–3.06)	<.001	2.09 (1.43–3.06)	<.001	2.09 (1.43–3.06)	<.001
65–69	2.75 (1.89–4.00)	<.001	2.87 (1.97–4.20)	<.001	2.87 (1.97–4.20)	<.001	2.89 (1.98–4.23)	<.001
70–74	2.85 (1.94–4.18)	<.001	3.14 (2.13–4.63)	<.001	3.14 (2.13–4.63)	<.001	3.17 (2.15–4.68)	<.001
≥75	2.52 (1.73–3.68)	<.001	2.85 (1.94–4.19)	<.001	2.85 (1.94–4.19)	<.001	2.87 (1.95–4.22)	<.001
Ethnicity [White]		.007		.012		.012		.008
Black	1.30 (1.05–1.62)	.017	1.30 (1.04–1.63)	.020	1.31 (1.05–1.63)	.018	1.34 (1.07–1.68)	.011
Asian	0.95 (0.76–1.20)	.683	0.94 (0.75–1.19)	.625	0.94 (0.75–1.19)	.629	0.95 (0.75–1.20)	.675
Mixed	1.63 (1.03–2.56)	.036	1.59 (1.00–2.53)	.049	1.59 (1.00–2.52)	.051	1.60 (1.00–2.54)	.048
Other	0.71 (0.44–1.15)	.161	0.75 (0.46–1.22)	.242	0.75 (0.46–1.23)	.255	0.77 (0.47–1.25)	.286
Deprivation Quintile [1 most deprived]		<.001		<.001		<.001		<.001
Quintile 2	0.93 (0.76–1.14)	.475	0.92 (0.75–1.12)	.403	0.92 (0.75–1.12)	.399	0.91 (0.74–1.11)	.364
Quintile 3	1.10 (0.88–1.38)	.410	1.08 (0.87–1.36)	.483	1.09 (0.87–1.36)	.472	1.08 (0.86–1.36)	.485
Quintile 4	1.36 (1.07–1.74)	.014	1.34 (1.05–1.72)	.021	1.35 (1.05–1.73)	.020	1.34 (1.05–1.73)	.020
Quintile 5 (least deprived)	1.66 (1.28–2.14)	<.001	1.62 (1.26–2.10)	<.001	1.64 (1.26–2.12)	<.001	1.62 (1.25–2.10)	<.001
Brief IPQ								
Question 1: Consequences	‐	‐	0.98 (0.95–1.01)	.147	0.98 (0.95–1.01)	.137	0.98 (0.95–1.01)	.115
Question 2: Timeline	‐	‐	1.02 (0.99–1.04)	.228	1.02 (0.99–1.04)	.231	1.01 (0.99–1.04)	.309
Question 4: Treatment control	‐	‐	1.03 (1.01–1.06)	.007	1.03 (1.01–1.06)	.007	1.03 (1.01–1.06)	.006
Question 6: Illness concern	‐	‐	1.06 (1.04–1.09)	<.001	1.06 (1.04–1.09)	<.001	1.06 (1.04–1.09)	<.001
NGS‐ES score	‐	‐	‐	‐	1.00 (0.99–1.01)	.485	1.00 (0.99–1.01)	.551
WEMWBS [High score]	‐	‐	‐	‐	‐	‐		.119
WEMWBS: Medium score	‐	‐	‐	‐	‐	‐	1.18 (1.01–1.39)	.042
WEMWBS: Low score	‐	‐	‐	‐	‐	‐	1.09 (0.87–1.37)	.460

*Note*: Step 1: region, gender, age, ethnicity, deprivation; Step 2: brief IPQ questions 1, 2, 4, 6; Step 3: NGS‐ES score; Step 4: WEMWBS. Nagelkerke *R* ^2^ = .056 (step 1); .077 (step 2); .077 (step 3); .078 (step 4); *R* ^2^ change = .022 (steps 1–4).

Abbreviations: [], Referent; CI, 95% confidence interval; IPQ, Brief Illness Perceptions Questionnaire; NGS‐ES, New General Self‐Efficacy Scale; OR, Odds Ratio (Exp(B) value); WEMWBS, Warwick‐Edinburgh Mental Wellbeing Scale.

Compared to participants living in North East London, the odds of starting the NHSDPP was significantly higher in those living in Cumbria (OR = 1.69; CI = 1.22–2.34; *p* = .002), and West Yorkshire (OR = 1.80; CI = 1.20–2.69; *p* = .004). As age increased, there was a significant increase in the odds of a person starting the NHSDPP when compared to the under‐40s. Compared with participants classified as White British/White, the odds of starting was 34% significantly higher in those from a Black ethnic group (OR = 1.34; CI = 1.07–1.68; *p* = .011). Compared with participants from more deprived areas (quintile 1), the odds of starting was higher in those who lived in more affluent areas (quintile 4—OR = 1.34; CI = 1.05–1.73; *p* = .020; quintile 5—OR = 1.62; CI = 1.25–2.10; *p* = <.001).

Multiple imputation was conducted to investigate the effect of missing data for all variables with more than 10% missing data (ethnicity, illness perception items, general self‐efficacy scores and uptake; see Appendix S3). In the logistic regression using imputed data, most statistically significant predictors in the complete case analysis remained significant in the imputed analysis (with no substantive changes in direction and magnitude). There were some minor changes with some of the demographic variables.

### Predictors of completion

Data were checked for independence from errors. For completion, the chi‐squared goodness‐of‐fit statistic showed there was no overdispersion (*χ* ^2^ (8): 4.963, *p* = .761). There was no evidence of collinearity between the predictor variables. Assumptions of linearity between the continuous predictors (illness perceptions, general self‐efficacy) and the logit of the outcome variable were met. The region with the lowest completion was used as the reference category (West Yorkshire: 1.1%, *n* = 9). Overall, the omnibus tests of model coefficients showed the model had a good fit against the null model (*χ* ^2^ (27): 194.482; *p =* <.001) but explained just 12.2% of variance (Nagelkerke *R* ^2^ = .122).

Higher general self‐efficacy scores (higher self‐efficacy) were associated with significantly decreased odds of completing the NHSDPP (OR = .97; CI = .96–.99; *p* = <.001) (Table 3). Compared with individuals with high mental wellbeing scores, those with low scores (low mental wellbeing) had 28% lower odds of completing (although not reaching significance) (OR = .72; CI = .50–1.05; *p* = .088). There were no associations between completion and medium mental wellbeing scores, or any illness perception items.

**TABLE 3:** Results of the binomial logistic regression model exploring predictors of completion of the NHSDPP.

	Step 1		Step 2		Step 3		Step 4	
	OR (95% CI)	*p*	OR (95% CI)	*p*	OR (95% CI)	*p*	OR (95% CI)	*p*
Region [West Yorkshire]		<.001		<.001		<.001		<.001
Cumbria	11.14 (4.42–28.07)	<.001	11.15 (4.42–28.10)	<.001	10.67 (4.23–26.91)	<.001	10.82 (4.29–27.32)	<.001
Herefordshire	19.41 (7.67–49.13)	<.001	19.49 (7.70–49.36)	<.001	17.50 (6.90–44.41)	<.001	17.43 (6.86–44.27)	<.001
Berkshire	8.89 (3.48–22.68)	<.001	8.93 (3.50–22.80)	<.001	8.49 (3.32–21.68)	<.001	8.48 (3.32–21.68)	<.001
South London	10.08 (4.02–25.23)	<.001	10.08 (4.03–25.26)	<.001	9.12 (3.63–22.88)	<.001	9.15 (3.64–22.96)	<.001
North East London	28.56 (10.62–76.80)	<.001	28.44 (10.57–76.53)	<.001	28.29 (10.50–76.22)	<.001	29.00 (10.75–78.21)	<.001
Gender [Male]: Female	1.01 (0.82–1.24)	.924	1.02 (0.83–1.25)	.866	1.01 (0.82–1.24)	.946	1.02 (0.83–1.26)	.843
Age [<40]		<.001		<.001		<.001		.001
40–44	0.94 (0.32–2.73)	.909	0.94 (0.33–2.74)	.916	0.97 (0.33–2.81)	.952	0.95 (0.33–2.78)	.932
45–49	1.12 (0.44–2.84)	.816	1.10 (0.43–2.81)	.837	1.12 (0.44–2.86)	.811	1.13 (0.44–2.90)	.793
50–54	1.30 (0.56–3.04)	.545	1.28 (0.55–3.01)	.568	1.31 (0.56–3.08)	.533	1.30 (0.55–3.06)	.547
55–59	1.54 (0.67–3.54)	.314	1.52 (0.66–3.50)	.329	1.53 (0.66–3.53)	.321	1.50 (0.65–3.47)	.345
60–64	1.85 (0.81–4.24)	.146	1.82 (0.79–4.18)	.160	1.87 (0.81–4.31)	.141	1.85 (0.80–4.26)	.151
65–69	2.05 (0.90–4.65)	.087	2.00 (0.88–4.55)	.101	2.00 (0.88–4.56)	.100	1.90 (0.83–4.36)	.128
70–74	3.13 (1.37–7.11)	.007	3.04 (1.33–6.94)	.008	3.13 (1.37–7.17)	.007	2.98 (1.30–6.84)	.010
≥75	2.10 (0.92–4.80)	.077	2.04 (0.89–4.69)	.093	2.05 (0.89–4.72)	.091	1.97 (0.85–4.53)	.113
Ethnicity [White]		.183		.198		.211		.181
Black	0.71 (0.50–1.03)	.072	0.71 (0.49–1.03)	.069	0.73 (0.51–1.06)	.094	0.72 (0.50–1.04)	.078
Asian	0.65 (0.43–0.98)	.038	0.66 (0.44–0.99)	.046	0.65 (0.43–0.99)	.043	0.64 (0.43–0.98)	.038
Mixed	0.77 (0.39–1.49)	.433	0.76 (0.39–1.48)	.419	0.71 (0.36–1.39)	.313	0.68 (0.35–1.35)	.271
Other	0.63 (0.24–1.71)	.366	0.64 (0.24–1.71)	.370	0.70 (0.26–1.88)	.474	0.72 (0.26–1.94)	.509
Deprivation Quintile [1 most deprived]		.094		.094		.050		.038
Quintile 2	0.91 (0.64–1.28)	.574	0.90 (0.64–1.28)	.564	0.89 (0.63–1.27)	.524	0.86 (0.61–1.23)	.414
Quintile 3	1.16 (0.81–1.65)	.430	1.14 (0.80–1.63)	.466	1.14 (0.80–1.64)	.466	1.12 (0.78–1.61)	.541
Quintile 4	1.45 (1.00–2.10)	.051	1.45 (1.00–2.10)	.051	1.49 (1.03–2.17)	.037	1.48 (1.02–2.15)	.041
Quintile 5 (least deprived)	1.23 (0.84–1.79)	.288	1.22 (84–1.78)	.300	1.28 (0.88–1.88)	.201	1.26 (0.86–1.84)	.239
Brief IPQ								
Question 1: Consequences	‐	‐	0.99 (0.95–1.03)	.570	0.99 (0.95–1.02)	.444	0.99 (0.95–1.02)	.448
Question 3: Personal control	‐	‐	1.03 (0.99–1.06)	.145	1.03 (0.99–1.06)	.109	1.03 (0.99–1.06)	.159
NGS‐ES score	‐	‐	‐	‐	0.98 (0.96–0.99)	<.001	0.97 (0.96–0.99)	<.001
WEMWBS [High score]	‐	‐	‐	‐	‐	‐		.045
WEMWBS: Medium score	‐	‐	‐	‐	‐	‐	1.10 (0.86–1.40)	.450
WEMWBS: Low score	‐	‐	‐	‐	‐	‐	0.72 (0.50–1.05)	.088

*Note*: Step 1: region, gender, age, ethnicity, deprivation; Step 2: brief IPQ questions 1, 3; Step 3: NGS‐ES score; Step 4: WEMWBS. Nagelkerke *R* ^2^ = .108 (step 1); .109 (step 2); .118 (step 3); .122 (step 4); *R* ^2^ change = .014 (steps 1–4).

Abbreviations: [], Referent; CI, 95% confidence interval; GS‐ES, New General Self‐Efficacy Scale; IPQ, Brief Illness Perceptions Questionnaire; OR, Odds Ratio (Exp(B) value); WEMWBS, Warwick‐Edinburgh Mental Wellbeing Scale.

Compared with those attending programmes in West Yorkshire, those attending programmes in other areas (Cumbria, Herefordshire, Berkshire, South London, North East London) had significantly higher odds of completing the NHSDPP, but with wide confidence intervals. Participants aged 70–74 years had approximately twice the odds of completing, compared to the under‐40s (OR = 2.98; CI = 1.30–6.84; *p* = .010). Compared with participants classified as White British/White, the odds of completing were 36% significantly lower in those from an Asian ethnic group (OR = .64, CI = .43–.98; *p* = .038). Compared with participants from more deprived areas (quintile 1), the odds of completing were significantly higher in those who lived in more affluent areas (quintile 4—OR = 1.48; CI = 1.02–2.15; *p* = .041).

Multiple imputation was conducted to investigate the effect of missing data for all variables included in logistic regression for completion (Appendix S4). Logistic regression using imputed data showed that some statistically significant predictors in the complete‐case analysis remained significant in the imputed analysis (all regions, age (70–74 years) and general self‐efficacy scores). Regarding the psychological variables, mental wellbeing (low score) was a significant predictor in the imputed analysis but not in the complete‐case analysis.

## DISCUSSION

We report the first study exploring whether illness perceptions, self‐efficacy and mental wellbeing predict uptake and/or completion of the NHSDPP, independent of participant socio‐demographics. Main findings are discussed, focusing on associations that were significant in logistic regression for both the original and imputed datasets.

### Uptake

Overall, illness perceptions related to treatment control and illness concern, mental wellbeing (among the psychological factors) and age and area of deprivation (among the demographic factors) were found to predict uptake. Self‐efficacy, on the other hand, did not predict uptake (not supporting hypothesis 2). This could mean that other external factors, such as accessibility and practicalities, could affect motivation and attendance, as suggested by other NHSDPP research (Begum et al., 2022).

Participants who saw treatment as being effective at controlling their prediabetes were more likely to start the NHSDPP, supporting hypothesis 1. This is in line with research that found non‐attenders of a cardiac rehabilitation programme were significantly less likely to feel their condition was controllable compared with attenders (French et al., 2006). This demonstrates the importance of ensuring that participants understand diabetes prevention programmes as effective treatments for prediabetes, and that their prediabetes is controllable.

Those participants who were more concerned about their prediabetes were also more likely to start the programme, which supports evidence that reports concern for personal health as a common reason for participating in diabetes prevention programmes (Parikh, Simon, et al., 2010). This indicates concern about prediabetes may be a motivator for people to attend diabetes prevention programmes. Both treatment control and illness concern can be explained using the Common‐Sense Model of Self‐Regulation (Leventhal, 1970; Leventhal et al., 1980, 1992). When individuals are faced with a health threat like prediabetes, cognitive representations and emotional responses like illness concern are generated in an attempt to make sense of their illness or health threat (Leventhal et al., 1992). This leads to the second stage, where in order to self‐regulate and cope with this, individuals will make efforts to find ways to manage these cognitions and emotions (Leventhal et al., 1992). These efforts will result in individuals participating in ‘common‐sense’ health behaviours such as attending a diabetes prevention programme that is offered to them after diagnosis. By having concern about prediabetes and seeing the diabetes prevention programme as appropriate treatment to control their prediabetes, this may encourage participant uptake.

Individuals with moderate mental wellbeing were more likely to start the NHSDPP compared with those with high or low mental wellbeing. One possible explanation could be that those with low mental wellbeing may have had low mood, felt depressed or perhaps were too anxious to start, as research has shown that individuals with depression are less likely to start lifestyle change programmes (Murray et al., 2012). Another possibility could be that those with low mental wellbeing may have had a high emotional response to their illness. It would be helpful to explore this further and determine whether a different approach would be more suitable for people with low levels of mental wellbeing (i.e., if you are feeling anxious or depressed you may not want to attend a group intervention). People with high mental wellbeing may not have felt the need to attend due to already feeling psychologically well and happy after making the required lifestyle changes. Another reason for the association between moderate wellbeing and uptake may be because of their concern about their prediabetes, which was also found to be a predictor of uptake in this study. This explanation was tested through further analysis where the relation between mental wellbeing and the illness perception concern item was examined. A Spearman's Rho correlation indicated a significant and moderate positive relationship between mental wellbeing and illness concern (rs = .57, *p* < .001, *N* = 3756). Before and/or during the initial assessment, it is important to ensure that participants understand the seriousness of prediabetes without instilling too much fear. The importance of attending diabetes prevention programmes and the support that will be offered regardless of whether one is already feeling psychologically well should be emphasized.

Older individuals were more likely to start the programme, which is in line with other diabetes prevention programme research that suggested older adults may have more time to take part due to having fewer work or caring responsibilities (Begum et al., 2022; Gray et al., 2016). As the age of onset for type 2 diabetes is decreasing, especially among certain ethnic groups (National Institute for Health and Care Excellence (NICE), 2011), it is important for diabetes prevention programmes to focus on improving uptake in younger adults. Those from the most affluent areas were more likely to start compared with those from the most deprived areas. Again, this accords with other diabetes prevention programme research (Gray et al., 2016; Valabhji et al., 2020). To allow diabetes prevention programmes to contribute to narrowing the health gap between the most and least deprived groups, there is a need to better engage those from more disadvantaged groups in such preventive health programmes.

### Completion

Overall, although mental wellbeing and illness perceptions were not found to predict completion (not supporting hypotheses 1 and 3), self‐efficacy (among the psychological factors), age (70–74 years) and region (among the demographic factors) were found to predict completion. Participants with higher self‐efficacy levels (confident in making lifestyle changes) were less likely to complete the NHSDPP. This finding did not support hypothesis 2 and contrasts with research on other lifestyle behaviour change programmes, which report that those with lower self‐efficacy were more likely to drop out (Jancey et al., 2007; Kampshoff et al., 2016). This could reflect that people with higher self‐efficacy felt that they already had the skills to make the required lifestyle changes and no longer needed the programme. In depth, qualitative research would be helpful to explore this finding further, as well as taking measures of variables at different time points to explore whether any of the psychological variables that emerged from the analysis changed as a result of the intervention. Such insight could inform the tailoring of programme content to be more relevant to those who feel (rightly or wrongly) that they already have the requisite skills to manage their prediabetes.

Similar to attendance, older adults (aged 70–74 years) were more likely to complete the programme than younger adults, again, a likely consequence of having fewer work or caring commitments, allowing more time to complete (Aujla et al., 2019). Other explanations also include that older adults are likely to be more adherent to treatment and other health behaviours when compared to younger adults, possibly due to prioritizing health more and having higher risk aversion (McLean et al., 2014; Parikh, Gupta, et al., 2010). Although individuals living in other regions were more likely to complete the NHSDPP compared to West Yorkshire, data from the National Diabetes Audit found that West Yorkshire had the highest uptake rates out of the regions included in this study (NHS Digital, 2020). This demonstrates that although uptake to the programme might be high, this does not necessarily mean high completion rates will be achieved. More research on this would provide further insights into other factors that could impact completion at a local level. Although the completion rate before analyses was low (14.7%), for the 2344 participants with sufficient data to be included in the logistic regression for completion, it was 22% (515 completers; 1829 non‐completers), which is in line with other NHSDPP research (Howarth et al., 2020; Valabhji et al., 2020).

### Strengths, limitations and future research

This is the first study to show the extent to which psychological factors influence uptake and completion of a diabetes prevention programme. Study strengths include the large sample, use of validated measures and the geographical spread.

Limitations are recognized. Firstly, these data were collected as part of the service provider's service evaluation, and a substantial proportion of uptake, ethnicity, illness perceptions and self‐efficacy data were missing. To account for this, a cautious approach was taken by using multiple imputation and focusing on associations that held in analysis of both original and imputed datasets. However, this meant certain demographic variables such as ethnicity (for both uptake and completion) and deprivation (for completion only) were not discussed due to only being significant in the complete‐case analysis. When compared with participants classified as White British/White, although those from a Black ethnic group were more likely to start the NHSDPP, those from an Asian ethnic group were less likely to complete. Other diabetes prevention programme research has also found that Asian and mixed ethnic groups had lower completion rates when compared to white groups (Valabhji et al., 2020). Also, those from the most affluent areas were more likely to complete the programme compared with those from the most deprived areas. This is consistent with other diabetes prevention programme research (Valabhji et al., 2020). Although these results indicate that the programme is somewhat reaching those who are at higher risk of developing type 2 diabetes such as those from black ethnic backgrounds, this is not the case for other ethnic groups or those from deprived backgrounds. Considering the importance of ethnicity and deprivation in the development of type 2 diabetes where individuals from South Asian, African‐Caribbean or black African family backgrounds, and those from lower socioeconomic groups are at a higher risk of developing type 2 diabetes (National Institute for Health and Care Excellence (NICE), 2011), further research is needed to improve uptake and completion among these specific groups. Future research could also explore how these demographic variables relate to the psychological variables explored in this study.

Secondly, it has been found that specific self‐efficacy is more predictive of health behaviour than general self‐efficacy (Bandura, 2006; Leganger et al., 2000). Future work could be undertaken to explore the influence of specific self‐efficacy (i.e. self‐efficacy for attending and/or completing the NHSDPP). Thirdly, in many cases confidence intervals were wide, indicating that estimates were not very precise. Finally, the majority of the variables included in the logistical regression models were categorical. Although, interpretability is an advantage of categorical data, some loss of precision is a limitation (Field, 2009).

## CONCLUSION

Overall, this study demonstrated the importance of psychological factors, particularly illness perceptions and mental wellbeing, in predicting uptake and self‐efficacy in predicting completion of a diabetes prevention programme. By programme organizers and clinicians taking these factors into consideration when recruiting participants and developing strategies to optimize uptake and retention, diabetes prevention programmes should become more effective and viable.

## AUTHOR CONTRIBUTIONS

**Sonia Begum:** Conceptualization; data curation; formal analysis; visualization; writing – original draft; writing – review and editing. **Rachel Povey:** Conceptualization; writing – review and editing. **Paul Chadwick:** Conceptualization; data curation; writing – review and editing. **Naomi Ellis:** Conceptualization; writing – review and editing. **Christopher Gidlow:** Conceptualization; visualization; writing – review and editing; formal analysis; data curation.

## FUNDING INFORMATION

This research did not receive any specific grant from funding agencies in the public, commercial, or not‐for‐profit sectors.

## CONFLICT OF INTEREST STATEMENT

None.

## ETHICS STATEMENT

Ethical approval was gained from Staffordshire University to conduct this study.

## PATIENT CONSENT STATEMENT

Informed consent was obtained from participants before data collection and consent was obtained to use their data for evaluation purposes.

## Supporting information


**Appendix S1.** Test for differences of IPQ item scores between those who did versus did not start the NHSDPP and between those who did vs. did not complete the NHSDPP.

**Appendix S2.** Test for differences of variables between those participants included in the logistic regression versus those excluded in the logistic regression for uptake.

**Appendix S3.** Pooled results from the binomial logistic regression for uptake using multiple imputed data.

**Appendix S4.** Pooled results from the binomial logistic regression for completion using multiple imputed data.

## Data Availability

Due to the nature of data and ethical approvals in place, data cannot be made available beyond the research team.
