# Supplementary material for: The effects of illness perceptions, self‐efficacy and mental wellbeing on uptake and completion of a diabetes prevention programme in England
Source: Br J Health Psychol. 2026 Jul 13;31(3):e70086. doi: 10.1111/bjhp.70086 (PMC13359074; doi:10.1111/bjhp.70086)
Supplement: Supplementary file 1 — Appendix S1. Test for differences of IPQ item scores between those who did versus did not start the NHSDPP and between those who did vs. did not complete the NHSDPP. [file BJHP-31-0-s002.docx]

*Appendix 1: Test for differences of IPQ item scores between those who did vs. did not start the NHSDPP and between those who did vs. did not complete the NHSDPP*

Mann-Whitney U-tests showed there to be statistically significant differences between those that took up the programme (i.e., started) and those that did not, in the IPQ items related to consequences, timeline, treatment control, and illness concern, and these IPQ items were used in the logistic regression model (Table 1).

*Table 1- Test for differences of IPQ item scores between those who did and did not start the NHSDPP*

|  |  | IPQ Items | | | | | | | |
| --- | --- | --- | --- | --- | --- | --- | --- | --- | --- |
|  | Uptake | Q1: Consequences | Q2: Timeline | Q3: Personal control | Q4: Treatment control | Q5: Identity | Q6: Illness concern | Q7: Coherence | Q8: Emotional response |
| N | Yes | 2,916 | 2,798 | 2,856 | 2,907 | 2,861 | 2,913 | 2,907 | 2,897 |
|  | No | 1,941 | 1,847 | 1,898 | 1,930 | 1,880 | 1,933 | 1,930 | 1,906 |
| Median score | Yes | 2.00 | 3.00 | 6.00 | 8.00 | 0.00 | 8.00 | 5.00 | 2.00 |
|  | No | 1.00 | 3.00 | 6.00 | 8.00 | 0.00 | 7.00 | 5.00 | 2.00 |
| Mann-Whitney *U* | - | 2729843.50 | 2423524.00 | 2708774.50 | 2452073.50 | 2682773.50 | 2543659.50 | 2801831.50 | 2675103.00 |
| z-score | - | -2.192 | -3.636 | -.034 | -4.737 | -.156 | -5.825 | -.073 | -1.879 |
| *p-*value (two-tailed) | - | 0.028 | <0.001 | 0.973 | <0.001 | 0.876 | <0.001 | 0.942 | 0.060 |

For completers, results from a Mann-Whitney U test showed there to be statistically significant differences between those that completed the programme and those that did not, in the IPQ items related to consequences and personal control, and these IPQ items were used in the logistic regression model (Table 2).

*Table 2- Test for differences of IPQ item scores between those who did and did not complete the NHSDPP*

|  |  | IPQ Items | | | | | | | |
| --- | --- | --- | --- | --- | --- | --- | --- | --- | --- |
|  | Completer | Q1: Consequences | Q2: Timeline | Q3: Personal control | Q4: Treatment control | Q5: Identity | Q6: Illness concern | Q7: Coherence | Q8: Emotional response |
| N | Yes | 636 | 600 | 613 | 613 | 622 | 626 | 621 | 626 |
|  | No | 2,280 | 2,198 | 2,243 | 2,232 | 2,239 | 2,287 | 2,286 | 2,271 |
| Median score | Yes | 2.00 | 3.00 | 7.00 | 8.00 | 0 | 8.00 | 5.00 | 2.00 |
|  | No | 2.00 | 3.00 | 6.00 | 8.00 | 0 | 8.00 | 5.00 | 3.00 |
| Mann-Whitney *U* | - | 686832.500 | 638122.000 | 649725.000 | 678387.500 | 670344.000 | 693284.500 | 694542.000 | 675925.500 |
| z-score | - | -2.120 | -1.228 | -2.104 | -.327 | -1.560 | -1.239 | -.830 | -1.933 |
| *p*-value (two-tailed) | - | 0.034 | 0.219 | 0.035 | 0.743 | 0.119 | 0.215 | 0.407 | 0.053 |
