# Supplementary material for: The effects of illness perceptions, self‐efficacy and mental wellbeing on uptake and completion of a diabetes prevention programme in England
Source: Br J Health Psychol. 2026 Jul 13;31(3):e70086. doi: 10.1111/bjhp.70086 (PMC13359074; doi:10.1111/bjhp.70086)
Supplement: Supplementary file 2 — Appendix S2. Test for differences of variables between those participants included in the logistic regression versus those excluded in the logistic regression for uptake. [file BJHP-31-0-s004.docx]

*Appendix 2: Test for differences of variables between those participants included in the logistic regression vs. those excluded in the logistic regression for uptake*

There were no statistically significant differences between those participants included in the logistic regression for uptake and those excluded, except for region, WEMWBS and the IPQ item related to timeline (Tables 1 & 2). There was a significant association between region and those excluded and included in the logistic regression (ꭕ2 (5, N=10,739) =643.178, p=<0.001) (Table 2). However, the association was weak (φ=.245) and accounted for just 6% of the variance (φ^2^=0.06). There was a significant association between WEMWBS and those excluded and included in the logistic regression (ꭕ2 (2, N=10,679) =11.601, p=0.003) (Table 2). However, the association was weak (φ=.033) and accounted for just 0.1% of the variance (φ^2^=0.001). The IPQ scores for timeline were not significantly different in those included and excluded in the logistic regression for uptake (*Mdn*=3.00) (Table 1).

*Table 1- Test for differences (Mann-Whitney U test) of variables between those included and excluded in the LR for uptake*

|  |  | LR variables | | | | |
| --- | --- | --- | --- | --- | --- | --- |
|  | Included in LR | Q1: Consequences | Q2: Timeline | Q4: Treatment control | Q6: Illness concern | GS-ES |
| N | Yes | 3,756 | 3,756 | 3,756 | 3,756 | 3,756 |
|  | No | 1,844 | 1,603 | 1,685 | 1,829 | 2,477 |
| Median score | Yes | 1.00 | 3.00 | 8.00 | 8.00 | 30.00 |
|  | No | 2.00 | 3.00 | 8.00 | 8.00 | 30.00 |
| Mann-Whitney *U* | - | 3389359.00 | 2845993.00 | 3101241.00 | 3422312.50 | 4581832.50 |
| z-score | - | -1.357 | -3.216 | -1.211 | -.227 | -1.010 |
| *p-*value (two-tailed) | - | 0.175 | 0.001 | 0.226 | 0.821 | 0.313 |

*Table 2- Test for differences (Multi-dimensional Chi-Square test) of variables between those included and excluded in the LR for uptake*

| Variables |  | Excluded in LR count (residual) | Included in LR count (residual) | Total (N) | Pearson Chi Square (ꭕ2) | df | Asymptotic Significance (2-sided) *p-*value | Phi (φ) |
| --- | --- | --- | --- | --- | --- | --- | --- | --- |
| **Region** |  | 6983 | 3756 | 10739 | 643.178 | 5 | <0.001 | 0.245 |
|  | North East London | 990 (116.1) | 354 (-116.1) | 1344 |  |  |  |  |
|  | Cumbria | 1011 (-2.7) | 548 (2.7) | 1559 |  |  |  |  |
|  | Herefordshire | 538 (-105.1) | 451 (105.1) | 989 |  |  |  |  |
|  | Berkshire | 327 (-255.6) | 569 (255.6) | 896 |  |  |  |  |
|  | South London | 2974 (-17.8) | 1627 (17.8) | 4601 |  |  |  |  |
|  | West Yorkshire | 1143 (265.2) | 207 (-265.2) | 1350 |  |  |  |  |
| **Gender** |  | 6901 | 3756 | 10657 | .516 | 1 | 0.473 | -0.007 |
|  | Women | 3811 (17.6) | 2047 (-17.6) | 5858 |  |  |  |  |
|  | Men | 3090 (-17.6) | 1709 (17.6) | 4799 |  |  |  |  |
| **Age** |  | 6968 | 3756 | 10724 | 15.426 | 8 | 0.051 | 0.038 |
|  | <40 | 313 (1.8) | 166 (-1.8) | 479 |  |  |  |  |
|  | 40-44 | 321 (4.6) | 166 (-4.6) | 487 |  |  |  |  |
|  | 45-49 | 484 (10.3) | 245 (-10.3) | 729 |  |  |  |  |
|  | 50-54 | 712 (-13.8) | 405 (13.8) | 1117 |  |  |  |  |
|  | 55-59 | 872 (-6.5) | 480 (6.5) | 1352 |  |  |  |  |
|  | 60-64 | 850 (-9.6) | 473 (9.6) | 1323 |  |  |  |  |
|  | 65-69 | 995 (-47.2) | 609 (47.2) | 1604 |  |  |  |  |
|  | 70-74 | 1092 (3.7) | 583 (-3.7) | 1675 |  |  |  |  |
|  | ≥ 75 | 1329 (56.8) | 629 (-56.8) | 1958 |  |  |  |  |
| **Ethnicity** |  | 4591 | 3756 | 8347 | 5.582 | 4 | 0.233 | 0.026 |
|  | White British or White | 2936 (13.2) | 2378 (-13.2) | 5314 |  |  |  |  |
|  | Black | 789 (-30.5) | 701 (30.5) | 1490 |  |  |  |  |
|  | Asian | 649 (14.3) | 505 (-14.3) | 1154 |  |  |  |  |
|  | Mixed | 105 (-5.6) | 96 (5.6) | 201 |  |  |  |  |
|  | Other | 112 (8.6) | 76 (-8.6) | 188 |  |  |  |  |
| **Deprivation quintile** |  | 6860 | 3756 | 10616 | 3.985 | 4 | 0.408 | 0.019 |
|  | 1 (most deprived) | 1593 (4.7) | 865 (-4.7) | 2458 |  |  |  |  |
|  | 2 | 1678 (12.8) | 899 (-12.8) | 2577 |  |  |  |  |
|  | 3 | 1356 (-37.8) | 801 (37.8) | 2157 |  |  |  |  |
|  | 4 | 1105 (16.8) | 579 (-16.8) | 1684 |  |  |  |  |
|  | 5 (least deprived) | 1128 (3.6) | 612 (-3.6) | 1740 |  |  |  |  |
| **WEMWBS** |  | 6923 | 3756 | 10679 | 11.601 | 2 | 0.003 | 0.033 |
|  | High | 2035 (42.8) | 1038 (-42.8) | 3073 |  |  |  |  |
|  | Medium | 3746 (-82.1) | 2159 (82.1) | 5905 |  |  |  |  |
|  | Low | 1142 (39.3) | 559 (-39.3) | 1701 |  |  |  |  |
