# Supplementary material for: The effects of illness perceptions, self‐efficacy and mental wellbeing on uptake and completion of a diabetes prevention programme in England
Source: Br J Health Psychol. 2026 Jul 13;31(3):e70086. doi: 10.1111/bjhp.70086 (PMC13359074; doi:10.1111/bjhp.70086)
Supplement: Supplementary file 3 — Appendix S3. Pooled results from the binomial logistic regression for uptake using multiple imputed data. [file BJHP-31-0-s001.docx]

*Appendix 3- Pooled results from the binomial logistic regression for uptake using multiple imputed data*

Some statistically significant predictors in the complete-case analysis did not remain significant in the imputed analysis, and these included: region (Cumbria & West Yorkshire), age (45-54 years), deprivation (quintile 4) and ethnicity (Black). Also, ethnicity (mixed) reached near significance in the complete-case analysis only. Region (Herefordshire), gender and ethnicity (Asian) were significant in the imputed analysis but not in the complete-case analysis, with region (Berkshire), deprivation (quintile 4) and age (50-54 years) reaching near significance. The odds of females starting the NHSDPP was 15% higher than males (OR= 1.15; CI= 1.04-1.26; *p*=0.005). Compared with participants classified as White British/White, the odds of starting the NHSDPP was lower in those from an Asian ethnic group (OR=0.76; CI=0.62-0.93; *p*=0.011).

|  | Step 1 | | Step 2 | | Step 3 | | Step 4 | |
| --- | --- | --- | --- | --- | --- | --- | --- | --- |
|  | OR (95% CI) | *p* | OR (95% CI) | *p* | OR (95% CI) | *p* | OR (95% CI) | *p* |
| Variables |  |  |  |  |  |  |  |  |
| **Region** [North East London]: Cumbria | 0.88 (0.73-1.07) | 0.198 | 0.91 (0.75-1.10) | 0.317 | 0.90 (0.74-1.10) | 0.297 | 0.90 (0.74-1.09) | 0.288 |
| Herefordshire | 0.81 (0.66-0.99) | 0.043 | 0.82 (0.66-1.01) | 0.059 | 0.81 (0.66- (1.00) | 0.048 | 0.80 (0.65-0.99) | 0.040 |
| Berkshire | 0.82 (0.67-0.99) | 0.044 | 0.82 (0.67-1.00) | 0.053 | 0.82 (0.67-1.00) | 0.046 | 0.82 (0.67-1.00) | 0.046 |
| South London | 1.01 (0.87-1.18) | 0.912 | 0.99 (0.85-1.17) | 0.932 | 0.99 (0.84-1.15) | 0.848 | 0.98 (0.84-1.15) | 0.824 |
| West Yorkshire | 1.05 (0.87-1.27) | 0.586 | 1.07 (0.88-1.30) | 0.491 | 1.07 (0.88-1.29) | 0.502 | 1.06 (0.88-1.29) | 0.521 |
| **Gender** [Men]: Women | 1.17 (1.07-1.29) | 0.001 | 1.15 (1.05-1.26) | 0.004 | 1.15 (1.04-1.26) | 0.005 | 1.15 (1.04-1.26) | 0.005 |
| **Age** [<40]: 40-44 | 0.93 (0.71-1.22) | 0.620 | 0.95 (0.73-1.25) | 0.733 | 0.96 (0.73-1.25) | 0.743 | 0.96 (0.73-1.26) | 0.750 |
| 45-49 | 1.12 (0.86-1.44) | 0.401 | 1.15 (0.88-1.50) | 0.302 | 1.15 (0.88-1.50) | 0.304 | 1.16 (0.89-1.50) | 0.285 |
| 50-54 | 1.24 (0.97-1.57) | 0.081 | 1.26 (0.99-1.61) | 0.062 | 1.26 (0.99-1.61) | 0.060 | 1.27 (1.00-1.62) | 0.054 |
| 55-59 | 1.43 (1.14-1.79) | 0.002 | 1.47 (1.17-1.84) | 0.001 | 1.47 (1.17-1.84) | 0.001 | 1.47 (1.18-1.85) | 0.001 |
| 60-64 | 1.46 (1.16-1.84) | 0.002 | 1.51 (1.19-1.91) | 0.001 | 1.50 (1.19-1.91) | 0.001 | 1.51 (1.19-1.91) | 0.001 |
| 65-69 | 1.94 (1.51-2.49) | <0.001 | 2.02 (1.57-2.60) | <0.001 | 2.02 (1.57-2.60) | <0.001 | 2.02 (1.56-2.60) | <0.001 |
| 70-74 | 1.92 (1.48-2.47) | <0.001 | 2.03 (1.57-2.62) | <0.001 | 2.03 (1.58-2.63) | <0.001 | 2.04 (1.58-2.64) | <0.001 |
| ≥75 | 1.66 (1.30-2.11) | <0.001 | 1.80 (1.40-2.32) | <0.001 | 1.80 (1.40-2.31) | <0.001 | 1.81 (1.41-2.32) | <0.001 |
| **Ethnicity** [White]: Black | 0.97 (0.83-1.13) | 0.703 | 0.93 (0.77-1.12) | 0.405 | 0.93 (0.77-1.12) | 0.409 | 0.94 (0.78-1.13) | 0.456 |
| Asian | 0.82 (0.69-0.98) | 0.029 | 0.76 (0.62-0.94) | 0.013 | 0.76 (0.62-0.94) | 0.013 | 0.76 (0.62-0.93) | 0.011 |
| Mixed | 1.13 (0.70-1.83) | 0.586 | 1.08 (0.64-1.80) | 0.757 | 1.07 (0.65-1.76) | 0.779 | 1.07 (0.65-1.77) | 0.764 |
| Other | 0.85 (0.61-1.18) | 0.317 | 0.83 (0.60-1.16) | 0.272 | 0.85 (0.61-1.18) | 0.311 | 0.85 (0.61-1.18) | 0.323 |
| **Deprivation Quintile** [1 most deprived]: Quintile 2 | 1.03 (0.91-1.16) | 0.660 | 1.02 (0.91-1.15) | 0.737 | 1.02 (0.90-1.15) | 0.745 | 1.02 (0.90-1.15) | 0.800 |
| Quintile 3 | 1.04 (0.90-1.19) | 0.598 | 1.04 (0.90-1.19) | 0.640 | 1.04 (0.90-1.19) | 0.637 | 1.03 (0.89-1.19) | 0.683 |
| Quintile 4 | 1.19 (1.02-1.38) | 0.028 | 1.17 (1.00-1.38) | 0.053 | 1.18 (1.00-1.38) | 0.049 | 1.17 (1.00-1.37) | 0.057 |
| Quintile 5 (least deprived) | 1.33 (1.14-1.54) | <0.001 | 1.31 (1.13-1.53) | <0.001 | 1.32 (1.13-1.53) | <0.001 | 1.31 (1.13-1.52) | <0.001 |
| **Brief IPQ** Question 1: consequences | - | - | 0.99 (0.96-1.02) | 0.627 | 0.99 (0.96-1.02) | 0.590 | 0.99 (0.96-1.02) | 0.569 |
| **Brief IPQ** Question 2: timeline | - | - | 1.02 (0.98-1.06) | 0.250 | 1.02 (0.98-1.06) | 0.252 | 1.02 (0.98-1.06) | 0.254 |
| **Brief IPQ** Question 4: treatment control | - | - | 1.04 (1.02-1.06) | 0.001 | 1.04 (1.02-1.06) | 0.001 | 1.04 (1.02-1.06) | 0.001 |
| **Brief IPQ** Question 6: illness concern | - | - | 1.06 (1.03-1.08) | <0.001 | 1.06 (1.03-1.08) | <0.001 | 1.06 (1.03-1.08) | <0.001 |
| **NGS-ES** score | - | - | - | - | 1.00 (0.99-1.00) | 0.258 | 1.00 (0.99-1.00) | 0.242 |
| **WEMWBS** [High score]: Medium score | - | - | - | - | - | - | 1.16 (1.05-1.28) | 0.004 |
| WEMWBS: Low score | - | - | - | - | - | - | 0.98 (0.86-1.12) | 0.748 |

Abbreviations: OR= Odds Ratio (Exp(B) value); CI= 95% confidence interval; IPQ=Illness perceptions questionnaire; NGS-ES= New general self-efficacy scale; WEMWBS= Warwick-Edinburgh mental well-being scale; []=Referent.
