# Supplementary material for: The effects of illness perceptions, self‐efficacy and mental wellbeing on uptake and completion of a diabetes prevention programme in England
Source: Br J Health Psychol. 2026 Jul 13;31(3):e70086. doi: 10.1111/bjhp.70086 (PMC13359074; doi:10.1111/bjhp.70086)
Supplement: Supplementary file 4 — Appendix S4. Pooled results from the binomial logistic regression for completion using multiple imputed data. [file BJHP-31-0-s003.docx]

*Appendix 4- Pooled results from the binomial logistic regression for completion using multiple imputed data*

Ethnicity (Asian) and deprivation (quintile 4) were significant predictors in the complete-case analysis but did not remain significant in the imputed analysis. Also, WEMWBS (low scores) and ethnicity (Black) reached near significance in the complete-case analysis only. Age (55-69 and ≥75 years), deprivation (quintile 2) and WEMWBS (low scores) were significant predictors in the imputed analysis but not in the complete-case analysis, with age (50-54 years), Asian and other ethnicity reaching near significance. Compared with individuals with high WEMWBS scores, those with low WEMWBS scores had 33% decreased odds of completing the NHSDPP (OR=0.67; CI=0.50-0.89; *p*=0.006).

|  | Step 1 | | Step 2 | | Step 3 | | Step 4 | |
| --- | --- | --- | --- | --- | --- | --- | --- | --- |
|  | OR (95% CI) | *p* | OR (95% CI) | *p* | OR (95% CI) | *p* | OR (95% CI) | *p* |
| Variables |  |  |  |  |  |  |  |  |
| **Region** [West Yorkshire]: Cumbria | 19.32 (9.76-38.25) | <0.001 | 19.33 (9.76-38.27) | <0.001 | 19.35 (9.78-38.31) | <0.001 | 19.59 (9.89-38.79) | <0.001 |
| Herefordshire | 35.57 (17.85-70.89) | <0.001 | 35.62 (17.87-70.99) | <0.001 | 35.06 (17.59-69.91) | <0.001 | 35.06 (17.58-69.91) | <0.001 |
| Berkshire | 18.35 (9.05-37.20) | <0.001 | 18.38 (9.06-37.27) | <0.001 | 18.48 (9.11-37.47) | <0.001 | 18.49 (9.11-37.51) | <0.001 |
| South London | 20.71 (10.54-40.70) | <0.001 | 20.68 (10.52-40.65) | <0.001 | 20.44 (10.40-40.19) | <0.001 | 20.44 (10.40-40.19) | <0.001 |
| North East London | 20.19 (9.82-41.52) | <0.001 | 20.11 (9.78-41.37) | <0.001 | 20.26 (9.85-41.66) | <0.001 | 20.49 (9.96-42.13) | <0.001 |
| **Gender** [Men]: Women | 0.95 (0.81-1.12) | 0.560 | 0.96 (0.82-1.12) | 0.596 | 0.95 (0.81-1.12) | 0.556 | 0.97 (0.83-1.14) | 0.685 |
| **Age** [<40]: 40-44 | 1.25 (0.55-2.86) | 0.591 | 1.25 (0.55-2.86) | 0.593 | 1.27 (0.56-2.91) | 0.566 | 1.27 (0.55-2.89) | 0.578 |
| 45-49 | 1.23 (0.58-2.61) | 0.584 | 1.22 (0.57-2.57) | 0.611 | 1.23 (0.58-2.60) | 0.591 | 1.24 (0.58-2.62) | 0.580 |
| 50-54 | 1.80 (0.91-3.56) | 0.089 | 1.78 (0.90-3.51) | 0.098 | 1.80 (0.91-3.55) | 0.092 | 1.81 (0.92-3.59) | 0.087 |
| 55-59 | 2.03 (1.05-3.95) | 0.037 | 2.00 (1.03-3.90) | 0.041 | 2.02 (1.04-3.94) | 0.038 | 2.03 (1.04-3.96) | 0.037 |
| 60-64 | 2.34 (1.21-4.54) | 0.012 | 2.31 (1.19-4.48) | 0.013 | 2.33 (1.20-4.52) | 0.012 | 2.32 (1.20-4.51) | 0.013 |
| 65-69 | 2.93 (1.53-5.64) | 0.001 | 2.88 (1.501-5.54) | 0.002 | 2.90 (1.50-5.58) | 0.001 | 2.79 (1.45-5.38) | 0.002 |
| 70-74 | 3.48 (1.81-6.70) | <0.001 | 3.41 (1.77-6.57) | <0.001 | 3.45 (1.79-6.64) | <0.001 | 3.31 (1.71-6.38) | <0.001 |
| ≥75 | 2.92 (1.52-5.64) | 0.001 | 2.86 (1.48-5.52) | 0.002 | 2.87 (1.49-5.54) | 0.002 | 2.79 (1.44-5.39) | 0.002 |
| **Ethnicity** [White]: Black | 0.83 (0.57-1.21) | 0.300 | 0.83 (0.57-1.21) | 0.314 | 0.83 (0.57-1.22) | 0.327 | 0.82 (0.56-1.20) | 0.285 |
| Asian | 0.75 (0.55-1.01) | 0.057 | 0.77 (0.57-1.04) | 0.083 | 0.77 (0.57-1.04) | 0.083 | 0.76 (0.56-1.03) | 0.072 |
| Mixed | 0.79 (0.44-1.41) | 0.417 | 0.79 (0.44-1.43) | 0.431 | 0.77 (0.43-1.37) | 0.367 | 0.75 (0.42-1.35) | 0.333 |
| Other | 0.39 (0.15-1.02) | 0.055 | 0.39 (0.15-1.05) | 0.061 | 0.41 (0.15-1.07) | 0.068 | 0.41 (0.15-1.07) | 0.068 |
| **Deprivation Quintile** [1 most deprived]: Quintile 2 | 0.79 (0.61-1.02) | 0.066 | 0.79 (0.61-1.02) | 0.065 | 0.79 (0.61-1.01) | 0.064 | 0.77 (0.59-0.99) | 0.040 |
| Quintile 3 | 0.86 (0.66-1.12) | 0.263 | 0.85 (0.65-1.12) | 0.244 | 0.85 (0.65-1.12) | 0.244 | 0.83 (0.64-1.09) | 0.187 |
| Quintile 4 | 1.12 (0.84-1.48) | 0.440 | 1.12 (0.84-1.48) | 0.441 | 1.12 (0.85-1.49) | 0.420 | 1.11 (0.84-1.47) | 0.472 |
| Quintile 5 (least deprived) | 0.88 (0.66-1.18) | 0.389 | 0.88 (0.66-1.17) | 0.376 | 0.89 (0.67-1.19) | 0.425 | 0.87 (0.65-1.16) | 0.340 |
| **Brief IPQ** Question 1: consequences | - |  | 0.99 (0.95-1.02) | 0.409 | 0.99 (0.95-1.02) | 0.350 | 0.99 (0.95-1.02) | 0.383 |
| **Brief IPQ** Question 3: personal control | - | - | 1.02 (0.99-1.06) | 0.183 | 1.02 (0.99-1.06) | 0.173 | 1.02 (0.99-1.06) | 0.232 |
| **NGS-ES** score | - | - | - | - | 0.99 (0.98-1.00) | 0.028 | 0.99 (0.98-1.00) | 0.013 |
| **WEMWBS** [High score]: Medium score | - | - | - | - | - | - | 1.07 (0.89-1.28) | 0.460 |
| WEMWBS: Low score | - | - | - | - | - | - | 0.67 (0.50-0.89) | 0.006 |

Abbreviations: OR= Odds Ratio (Exp(B) value); CI= 95% confidence interval; IPQ=Illness perceptions questionnaire; NGS-ES= New general self-efficacy scale; WEMWBS= Warwick-Edinburgh mental well-being scale; []=Referent.
